# The Use of Coumarins as Environmentally-Sensitive Fluorescent Probes of Heterogeneous Inclusion Systems

**DOI:** 10.3390/molecules14010210

**Published:** 2009-01-06

**Authors:** Brian D. Wagner

**Affiliations:** Department of Chemistry, University of Prince Edward Island, 550 University Ave, Charlottetown, PE C1A 4P3 Canada

**Keywords:** Coumarins, Fluorescence spectroscopy, Fluorescent probes, Heterogeneous systems, Host-guest inclusion.

## Abstract

Coumarins, as a family of molecules, exhibit a wide range of fluorescence emission properties. In many cases, this fluorescence is extremely sensitive to the local environment of the molecule, especially the local polarity and microviscosity. In addition, coumarins show a wide range of size, shape, and hydrophobicity. These properties make them especially useful as fluorescent probes of heterogeneous environments, such as supramolecular host cavities, micelles, polymers and solids. This article will review the use of coumarins to probe such heterogeneous systems using fluorescence spectroscopy.

## Introduction

**Figure 1:**
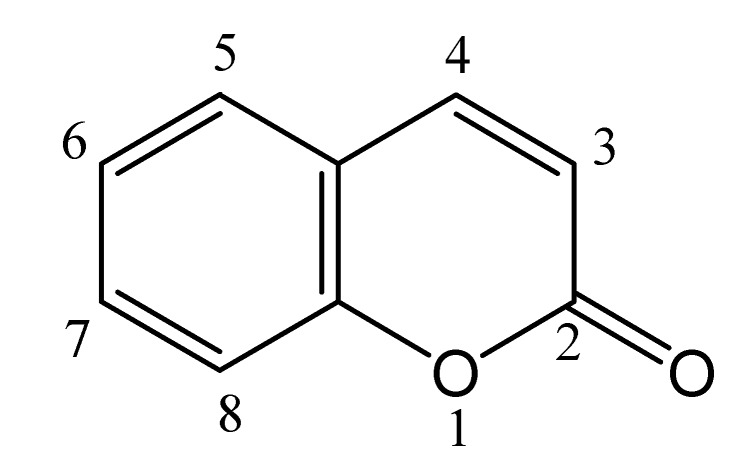
The chemical structure and numbering scheme of coumarin.

Coumarins, or benzo-α-pyrones, are a very large and important family of compounds. Their defining structure consists of fused pyrone and benzene rings, with the pyrone carbonyl group at position 2 [1]; this structure is illustrated in Figure 1 for the coumarin parent molecule (IUPAC name: 2*H*-chromen-2-one, and also known as 1-benzopyran-2-one). Coumarins are widely occurring in nature, with coumarin itself first isolated in 1820 from a specific variety of bean, and many other coumarin derivatives found in a wide range of plants [1]. As a group, coumarins exhibit interesting fluorescence properties, which include a high degree of sensitivity to their local environment, including polarity and viscosity. This sensitivity has led to their widespread application as sensitive fluorescent probes of a wide range of systems, including homogeneous solvents and mixtures, and heterogeneous materials; the latter is the focus of this article. Specifically, the purpose of this review is two-fold: 1) to provide a detailed review of the use of coumarin fluorescence to probe the nature and properties of heterogeneous materials and systems, and 2) to provide a guide to current and future researchers studying heterogeneous and supramolecular systems to the utility of and information provided by coumarins as fluorescent probes.

Numerous fluorescent coumarin derivatives have been reported, with a wide range of polarity, pH, viscosity, and other sensitivities, and varying underlying photophysical mechanisms for the observed fluorescence properties. Furthermore, thousands of papers involving some aspect of coumarin chemistry or spectroscopy have been published. Therefore, a comprehensive review of all coumarin derivatives, fluorescence properties and applications is beyond the scope of this article. Instead, the article begins with an overview of coumarin fluorescence properties using two representative groups of specific coumarin derivatives. A detailed review of the use of the fluorescence of included coumarins to study specific types of heterogeneous chemical systems and media is then presented. The use of coumarin fluorescence to probe proteins and other biochemical and biological systems will not be covered. In addition, the extensive use of coumarins as covalently attached fluorescent labels and structural components, or to generate fluorescent derivatives, will also not be reviewed, nor will the use of coumarins as fluorescent laser dyes. Thus, the scope of the review will be limited to the use of discrete coumarin fluorescent probe molecules which become included in the cavities or internal structure of hosts, discrete organized chemical structures in solution (such as micelles and polymers) or solid materials.

## Coumarin Photophysics

In this section, the defining features of coumarins as fluorescent probes is discussed. In particular, the mechanism for the high degree of polarity- and viscosity-sensitivity of the fluorescence of this family of compounds is described, using specific representative coumarin derivative examples. As stated above, the purpose of this section is to provide a representative overview of coumarin photophysics, in regards to their usefulness as environmentally-sensitive fluorescent probes. In addition, specific coumarins and effects are discussed in later sections dealing with applications to specific heterogeneous systems.

In order to describe the fluorescence of coumarin probes, there are four experimental properties of interest, which can exhibit significant changes within heterogeneous systems. These are the wavelength of maximum fluorescence intensity (*i.e.* the wavelength of the peak of the spectrum), λ_F,max_ (alternatively the frequency of maximum intensity, ν_F,max_), the fluorescence emission intensity at a particular wavelength, I_F_, the fluorescence quantum yield, N_F_, and the fluorescence lifetime, τ_F_. The value of λ_F,max_ is indicative of the energy gap between the fluorescent and ground singlet states, and can undergo significant blue-shifting (to shorter wavelength/higher frequency and energy) or red-shifting (to longer wavelength/lower frequency and energy) in response to the local environment. The value of I_F_ is indicative of the intensity of the fluorescence of the particular sample, whereas the value of N_F_ is a measure of the efficiency of the fluorescence of the probe in this sample as a relaxation pathway, relative to all relaxation pathways, including nonradiative decay. (The value of N_F_ is related to the integrated intensity I_F_ taken over the entire spectrum, relative to that of a fluorescent standard). The value of τ_F_ is a measure of the lifetime of the excited state, and depends on all of the deactivation pathways available to the excited state. Both N_F_ and τ_F_ are related to the rate constants for radiative (k_r_) and nonradiative (k_nr_) decay: N_F_ = k_r_ /(k_r_ + k_nr_); τ_F_ = 1/(k_r_ + k_nr_). The values of all three properties I_F_, N_F_ and τ_F_ can be significantly increased or decreased upon changing the local environment of the coumarin probe; the mechanisms for this will be discussed throughout this section.

A detailed spectroscopic study of coumarin itself was published in 1970 by Song and Gordon [2]. They measured fluorescence and phosphorescence spectra and lifetimes in both polar and nonpolar solvents at 77 K. They assigned the fluorescence emission to a ^1^(π,π*) excited state, and observed a large red-shift of 30 nm in nonpolar as compared to polar solvent. Thus, the significant solvatochromism of coumarin fluorescent probes has been known for almost 40 years. 

**Figure 2:**
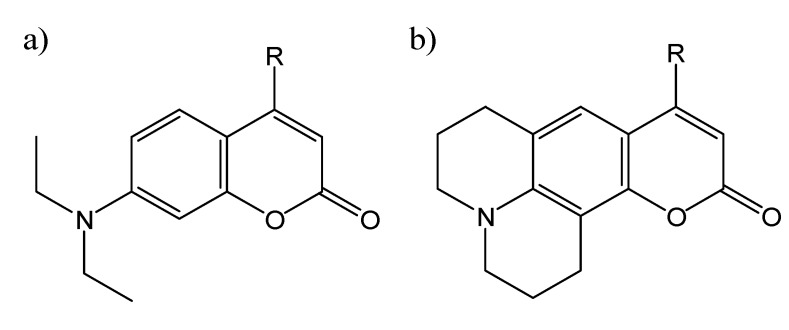
The chemical structure of some commonly-used 7-aminocoumarin fluorescent probes. a) R=CH_3_: C1, also known as C460; R=CF_3_: C1F, also known as C35, C152A and C481; b) R=CH_3_: C102, also known as C480; R=CF_3_: C6F, also known as C153 and C540A.

7-Aminocoumarins such as 4-methyl-7-diethylaminocoumarin (**C1**, shown in Figure 2a) are arguably the most important subset of coumarins, and have been the focus of intense study [3,4,5,6,7,8,9,10,11,12,13,14,15,16,17,18,19] and wide-spread applications as fluorescent probes. The photophysics of 7-aminocoumarins will thus be described in some detail, as a representative group. The chemical structures of some commonly-used 7-aminocoumarin fluorescent probes are shown in Figure 2. It should be noted that different authors sometimes use different coumarin numbers to describe the same coumarin derivative, thus all relevant numbers will be indicated with each coumarin structure shown. (Throughout this review, coumarin derivatives for which the chemical structure is shown in one of the figures will be indicated in bold.)

Jones *et al.* [4] presented an early study on the effect of solvents on three 4-trifluoromethyl substituted 7-aminocoumarins, **C1F** (Figure 2a), **C6F** (Figure 2b), and C8F. They observed a very strong red-shift in polar solvents, and were able to correlate ν_F,max_ with the solvent polarity-polarizability parameter π*, as well as the hydrogen bonding parameter α. They also observed a significantly reduced fluorescence quantum yield N_F_ for the non-rigid coumarin **C1F** with increasing polarity, which they attributed to an increased nonradiative decay rate k_nr_, via formation of a twisted intramolecular charge-transfer (TICT) state. Jones *et al.* then followed up with an expanded study [5], which included 11 different 7-aminocoumarin laser dyes, and reported that hydrogen bonding is the major factor controlling TICT formation in each case, and that this effect explains the observed solvatochromism. In fact, TICT formation [20] plays the defining role in 7-aminocoumarin fluorescence properties [4,5,6,7,8,9,10,11,12,14,16,18,20], through increased TICT nonradiative decay in polar media. As a result of the amino group, 7-aminocoumarins also exhibit pH-dependent fluorescence. Patalakha *et al.* reported on the acid-base properties of a series of 7-diethylaminocoumarins with various aromatic substituents [7] or fluorine [8] at position 3, and observed very large blue shifts in the fluorescence emission of the protonated as compared to the neutral form of these probe molecules. Abdel-Mottaleb [9] studied the photophysics of both flexible and rigid 7-aminocoumarin derivatives as a function of viscosity in aqueous glycerol solutions. They measured the fluorescence depolarization rate, and correlated this with the calculated free volume fraction of the medium. They used these results to propose the use of these 7-aminocoumarins as fluorescent probes of both fluidity and polarity of the local medium. Yip *et al.* [10] measured the fluorescence lifetimes of coumarins **C1** and **C102** (Figure 2b) in a number of polar solvents, and obtained two- and three-exponential decays. They attributed these results, as well as differences in decay-associated fluorescence spectra, to an irreversible two-state solvation model. 

**Figure 3:**
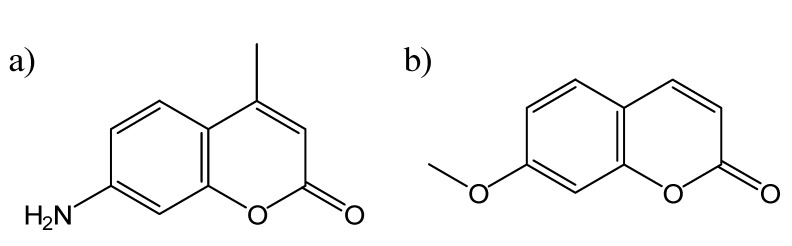
The chemical structures of a) coumarin derivative C120; b) 7-methoxycoumarin (7MC).

There has been some controversy on the exact role that hydrogen bonding plays in the formation of TICT states in protic solvents. López Arbeloa *et al.* [11] studied the photophysics of a number of 7-aminocoumarin derivatives and invoked specific hydrogen bonding between the coumarins and solvent to explain the observed solvent dependence. Królicki *et al.* [13] also investigated the role of hydrogen bonding, this time in the case of the rigid 7-aminocoumarin **C153** (Figure 2b), in mixed solvent systems, and observed preferential solvation in the excited state, but not the ground state. They also observed an unusual dependence of the fluorescence quantum yield on the mole fraction of methanol in methanol:toluene mixed solvent, and attributed this to specific hydrogen bonds between methanol and the coumarin probe. More recently, Moog *et al.* [15] extended the study of coumarin solvatochromism of coumarins **C1**, **C120** (Figure 3a), C151, C152 and **C153** by comparing three different models for treating solvent effects. They found that the multi-parameter Kamlet-Taft equation gave the best correlation for all of these coumarins, and further concluded that the effect of hydrogen bonding to solvent was a result of the increased field produced by the dipole moment of the hydrogen-bonding solvent, and not the hydrogen bonding interaction itself, in contrast to the results described above of López Arbelo *et al.* [11] and Królicki *et al.* [13]. However, Dahiya *et al.* [16] reported the effects of protic solvents on the coumarins C152 and **C481** (Figure 2a), and concluded that the solute-solvent H-bonding interactions directly stabilize the TICT states. Most recently, Barik *et al.* [18] published a detailed study on the evidence for TICT-mediated nonradiative decay of coumarin **C1** in high polarity protic solvents. They were able to correlate the observed Stokes shift with the solvent polarity, described using the parameter Δf. Furthermore, they saw no evidence for TICT in highly polar aprotic solvents, emphasizing the role of hydrogen bonding to the solvent. They also observed an exponential increase in the nonradiative decay rate in solvents with polarity Δf > 0.28, indicating the onset of an additional nonradiative deactivation pathway. It is clear from these studies that in the case of water, which is the predominant solvent of choice for studying heterogeneous systems in solution, hydrogen bonding plays a significant role in the formation of TICT states in 7-aminocoumarins, as will the polarity differences within the heterogeneous medium as compared to the bulk water solvent. 

Other specific studies of 7-aminocoumarin photophysics have been reported, including the effect of substituents and concentration [12], the observation of multiple emissions from 7-aminocoumarins with heterocyclic substituents [14], and photochemical transformations upon UV irradiation [17]. In addition, Sharma *et al.* [19] determined the dipole moments of a number of 7-aminocoumarin dyes, both experimentally and theoretically, and found that in all cases, the dipole moment was much larger in the excited state.

It is clear from all of these studies that 7-aminocoumarins, as a group, are very sensitive fluorescent probes for the study of local environments within heterogeneous media. They have been used significantly for this purpose, as described in the following sections. The fluorescence of these probes is strongly dependent on the polarity, hydrogen bonding ability, pH and microviscosity or rotational hindrance of their local environment, and this dependence varies with the specific 7-aminocoumarin derivative used. In general, as a result of TICT state formation in polar solvents, the fluorescence emission of 7-aminocoumarins is seen to red-shift and decrease in intensity as the polarity of the medium is increased.

Another useful subset of coumarin probes is the 7-alkoxycoumarins [21,22], such as 7-methoxycoumarin (**7MC**, the structure of which is shown in Figure 3b). These coumarins exhibit a different polarity-dependent fluorescence than do 7-aminocoumarins: their fluorescence intensity *increases* with increasing polarity of the medium, but with negligible spectral shift [21,22]. For example, the value of N_F_ for 7-methoxycoumarin is 0.51 in aqueous buffer (*i.e.* over half of the excited molecules relax by emitting a photon), but drops to 0.033 in methanol [22]; however the wavelength of maximum emission only changes from 324.7 to 322.6 nm. This solvent dependence is a result of a completely different mechanism than the TICT formation described above for 7-aminocoumarins, and involves the changing of the energy of the ^1^(ππ*) fluorescent state relative to the closely lying first triplet ^3^(nπ*) state [21]. In nonpolar solvent, the ^1^(ππ*) state lies just above the ^3^(nπ*) state, so that the rate of nonradiative intersystem crossing (ISC) is very efficient, and N_F_ is correspondingly low. However, in polar solvent, the ^1^(ππ*) energy is lowered below that of the triplet state, greatly decreasing the efficiency of ISC, and thus N_F_ increases significantly. This effect of polarity on emission intensity is the exact opposite of that observed with 7-aminocoumarins.

**Figure 4:**
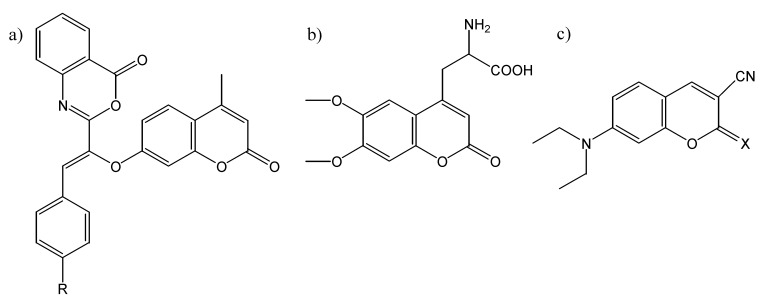
The chemical structure of a) a pair of specifically-designed viscosity-dependent coumarin fluorescent probes (R = OH or CO_2_CH_3_); b) a coumarin-based amino acid probe; c) some iminocoumarins (R = NH or N(CO)OCH_2_CH_3_).

Many other coumarin derivatives have been designed for specific fluorescence properties or sensitivities, such as cyanocoumarins [23] and 7-hydroxycoumarin-hemicyanine hybrids [24], both of which exhibit emission in the red region of the spectrum, and the pair of highly substituted coumarins shown in Figure 4a, which show fluorescence dependence solely on viscosity, rather than polarity [25]. Some other useful coumarin derivatives with specific applicability that have been prepared and studied include 3-(2’-benzimidazolyl) coumarins [26], the coumarin-based amino acid shown in Figure 4b [27], various iminocoumarins (such as those shown in Figure 4c) [28], and various biscoumarins [29,30]. 

In addition to the experimental spectroscopic studies of coumarins as described above, there have also been a number of useful theoretical studies of coumarin derivatives [9,19,31,32,33], in which the energies and electronic configurations of the excited singlet and triplet states have been calculated, in order to help to understand coumarin photophysics. The computational and theoretical approaches used include SCF-CI [9], PM3 [19], AM1 [31], PPP [32] and MM2 calculations [33]. 

## Coumarins as Fluorescent Guests Included in Molecular Hosts

**Figure 5:**
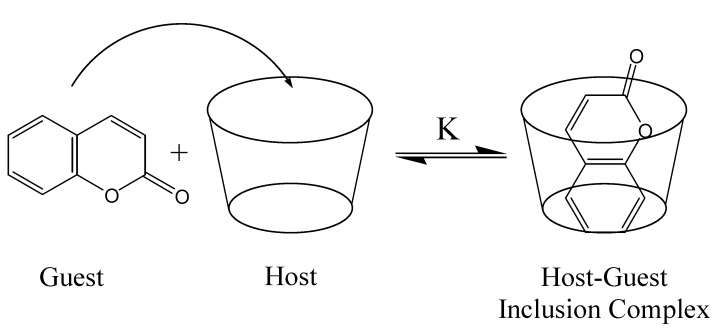
A representation of the inclusion of a coumarin guest inside a host cavity, forming a host-guest inclusion complex.

Host-guest inclusion complexes are formed when a small *guest* molecule becomes encapsulated within the internal cavity of a larger, cage-like *host* molecule. Such complexes represent one of the simplest examples of a supramolecular system, as the complex is held together only by non-covalent forces. Because of the lack of covalent binding between the host and guest, this complexation is a dynamic phenomenon, and equilibrium is established in solution between the complex and the free host and guest, as illustrated in Figure 5. The value of the binding constant, K, is the most important measurable property of the host-guest complex, and its magnitude is indicative of the total driving forces for inclusion. In order for a significant concentration of the host-guest complex to be obtained, the rate of entrance into the cavity (k_in_) must be significantly larger that the rate of exit (k_out_); K = k_in_/k_out_. The phenomenon of host-guest inclusion is an important aspect of the recent and growing field of supramolecular chemistry, and has found widespread and important applications, as discussed below for individual families of hosts.

Host-guest inclusion complexes are usually formed in aqueous solution, as this maximizes the difference in local polarity between the relatively nonpolar internal cavity of the organic host molecule and the bulk solvent, maximizing the *hydrophobic effect* as a driving force for inclusion of hydrophobic guests. Thus, the guest will experience a significant lowering of the polarity of the local environment upon inclusion into the host cavity. Furthermore, the guest will be in the much more confining and restrictive cavity as opposed to being free in solution, so that guest intramolecular rotations will be expected to be significantly hindered, depending on the size and shape of the cavity. For both of these reasons, coumarins are ideal fluorescent guests to probe the nature and binding capacity of such host molecules, as both the polarity and the constriction will greatly affect the formation of TICT states, and the polarity will change the relative energy levels, all of which result in significant and easily measurable changes in the coumarin probe fluorescence.

### Cyclodextrins

**Figure 6:**
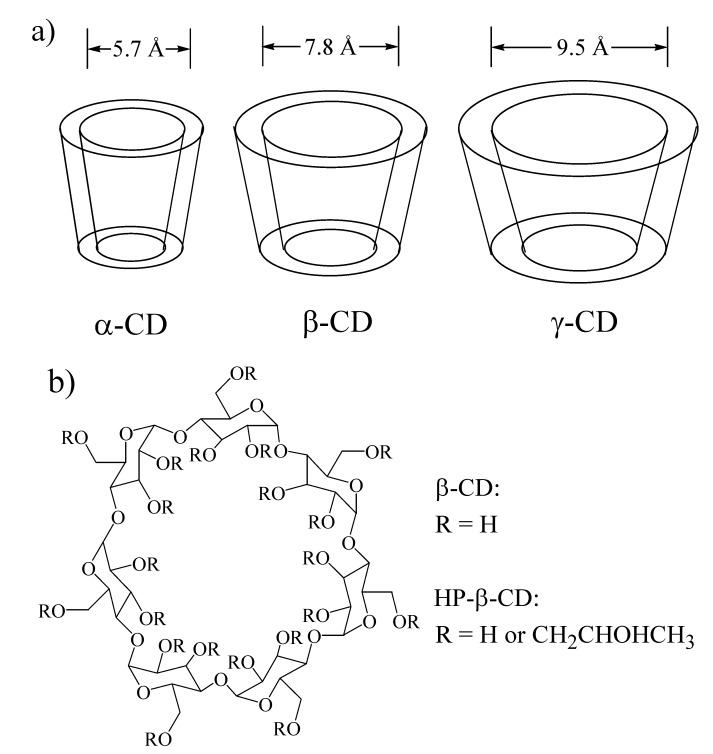
a) Molecular bucket depictions and cavity sizes of α-, β-, and γ-CD; b) chemical structure of native and HP-modified β-CD.

Cyclodextrins are cyclic oligosaccharides of glucopyranose, which through intramolecular hydrogen bonding form truncated cone-shaped structures with large internal cavities in aqueous solution [34]. The presence of the large, internal cavity makes cyclodextrins, also referred to as “*molecular buckets*”, excellent hosts for the inclusion of a wide range of neutral and ionic guests [34], and they are by far the most widely studied and utilized molecular hosts [34,35]. As shown in Figure 6a, there are three “native” cyclodextrins, α-, β- and γ-CD, which consist of six, seven, and eight gluocopyranose units, respectively, and hence have very different cavity sizes. The chemical structure of β-CD is shown in Figure 6b; also illustrated is the presence of the three hydroxyl groups per glucopyranose unit, which allow CDs to be readily chemically modified. The relative ease of preparing modified CDs, with specific targeted properties, has also contributed to their huge popularity as molecular hosts. 

There is a relatively long and rich history of the use of the fluorescence of coumarins to investigate CDs, with the first such study reported by Takadate *et al.* in 1983 [36]. They studied five different 7-substituted 4-methylcoumarins included in β-CD, and found that the fluorescence was enhanced and blue shifted for the 4-hydroxy- and 7-aminocoumarins, but that the fluorescence was quenched for 7-methoxy and 7-ethoxycoumarin. In all cases, the observed fluorescence effects were interpreted in terms of the relative polarity of the CD cavity relative to bulk water; which the authors determined to be slightly more polar than ethanol solvent, based on the measured fluorescence maxima. The authors were able to use the change in fluorescence as a function of added CD concentration to obtain the binding constant K, which ranged widely from 80 M^-1^ in the case of 7-methoxy-4-methylcoumarin to 893 M^-1^ in the case of 7-dimethylamino-4-methylcoumarin. This range in K values illustrates the impact that guest size, shape and properties (such as polarity) can have on the strength of the binding with CDs. This work was followed shortly thereafter in 1985 by a report of Scypinski and Drake [37] on the inclusion of the rigid 7-aminocoumarin derivative **C540A** (Figure 2b) in β- and γ-CD. Significant enhancement of **C540A** fluorescence was observed in both CDs (slightly larger in γ-CD), with a blue shift of 10 nm. Both observations were attributed to the formation of 1:1 CD: guest inclusion complexes, and corresponding decreased polarity within the CD cavity relative to that of water. No change in **C540A** fluorescence was observed in the presence of α-CD, indicating that the cavity of this CD is too small to accommodate this large coumarin guest. The values of the binding constant, K, obtained were quite small, only 54 M^-1^ for β-CD at 20 °C. Interestingly, two types of complexes were obtained, a “normal” and an “inverted” complex, depending on the conditions used to prepare them, with the **C540A** having opposite orientations within the cavity in the two types of complex. While the “normal” complex showed the fluorescence enhancement described, the “inverted” complex actually exhibited reduced fluorescence, due to enhanced quenching of the exposed guest. Hydrogen bonding between the host and guest was proposed to occur in the “normal” complex. Two other studies of the inclusion of 7-aminocoumarins in CDs have subsequently been reported. Bergmark *et al.* [38] showed that the co-inclusion of organic solvents with coumarins **C1** and **C6F** in β- and γ-CD resulted in even greater enhancement of the coumarin fluorescence. They proposed that this additional enhancement occurred due to the displacement of CD cavity water by these organic solvent molecules; these water molecules directly quenched the coumarin fluorescence in solution or in CDs in the absence of organic co-solvents. However, under identical conditions, the fluorescence of coumarin **C1F** was found to *decrease*, illustrating the tremendous difference in probe fluorescence properties which can be obtained with only minor differences in the coumarin structure (in this case, replacement of a CH_3_ by a CF_3_). The presence and role of cavity water elucidated by this coumarin study is a very important property of CD cavities, and has significant effects on their host properties. Nowakowska *et al.* [39] showed that inclusion of 7-amino-4-methylaminocoumarin **C120** into both β- and γ-CD provided significant photostabilization of this coumarin dye, illustrating a very useful application of CDs as guest stabilizers [35].

Other types of coumarins have also been used to study native CDs. Al-Kindy *et al.* [40] showed that both α- and β-CD significantly enhanced the fluorescence of coumarin-6-sulfonyl chloride amino acid derivatives, and that a stable 2:1 β-CD:guest inclusion complex was formed, with a very large overall 2:1 binding constant of K = 4.7 × 10^7^ M^-2^ for the alanine derivative. The value of the binding constant was found to depend strongly on the size of the coumarin derivative, and the possible interactions with the CD host, once again illustrating the importance of the size and fit match of guests with the CD cavity. They proposed the use of these CD-coumarin complexes as fluorescent sensors for amino acids. 

**Figure 7:**
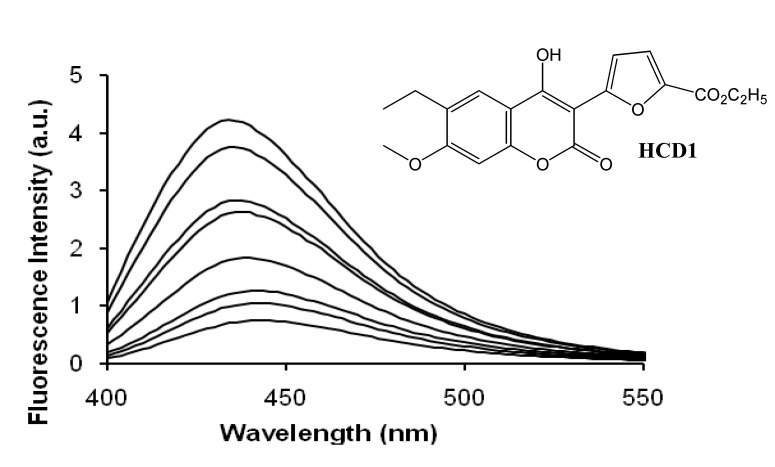
Structure of **HCD1** and the fluorescence spectrum of its solution (1 × 10^-5^ M) in deionized water (1.3% ethanol) in the presence and absence of β-CD. From bottom to top: [β-CD] = 0, 1 × 10^-4^, 2 × 10^-4^, 1 × 10^-3^, 2 × 10^-3^, 4 × 10^-3^, 7 × 10^-3^, and 1 × 10^-2^ M. Spectra reproduced with permission from Figure 3 in Reference [41]. Copyright 2001 American Chemical Society.

Dondon and Fery-Forgues [41] studied the effect of β-CD on two 4-hydroxycoumarins substituted with heterocyclic substituents in the 3 position, **HCD1** (structure shown in Figure 7) and HCD2. They found significant fluorescence enhancement upon CD inclusion, which they attributed to the constrictive effect of the CD cavity. The fluorescence spectrum of HCD1 as a function of added β-CD is also shown in Figure 7; this result is an excellent example of the fluorescence enhancement effect of host inclusion on many coumarin guests, including 7-aminocoumarins as well as the 7-hydroxycoumarin derivative shown. Significant 1:1 binding constants of 340 and 700 M^-1^ were obtained for the two derivatives; however, a much lower binding constant of 81 M^-1^ was obtained for the 4-hydroxycoumarin in the absence of the heterocyclic substituent, illustrating the lower affinity of CDs for phenolic guests. 

A number of studies have been reported using coumarin guests to study dynamics within native CD cavities [42,43,44]. Vajda *et al.* [43] used femtosecond fluorescence upconversion and time-correlated single photon counting techniques to study the solvation dynamics of two coumarins, **C480** (Figure 2b) and **C460** (Figure 2a), in aqueous solution and included within the cavity of γ-CD, which is large enough to co-include a small number of water molecules. Solvation of **C480** was found to occur on the fs timescale in pure water, but on the ps to ns timescale within γ-CD, again illustrating the highly restrictive nature of CD cavities. More recently, Bhattacharyya’s group [44] studied the temperature-dependence of the anisotropy decay of **C153**, once again in γ-CD, the largest native CD cavity. Interestingly, they found that the **C153** guests served as linkers between γ-CD hosts, generating linear “nanotube aggregates”, resulting in very large steady-state and residual anisotropies. They further found a strong temperature dependence of the **C153** solvation time within the CD nanotubes, which they attributed to a dynamic exchange between free and cavity-bound water molecules; this dynamic exchange of cavity water is an important feature of aqueous CD hosts.

**Figure 8:**
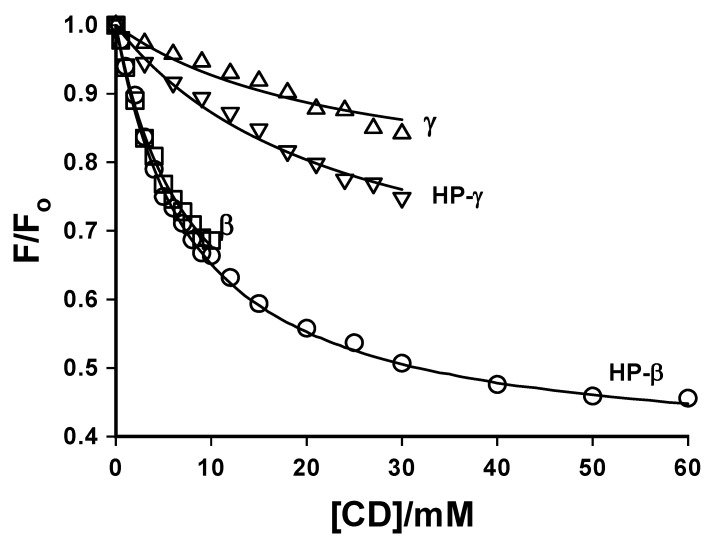
The effect of cyclodextrin concentration on the relative total fluorescence (F/F_o_) of 7-methoxycoumarin (λ_ex_ = 320 nm) for various cyclodextrins: ☐ β-CD, ◯ HP-β-CD, **Δ** γ-CD, **∇** HP-γ-CD; the solid lines show the fit to a 1:1 host:guest complex model. Reproduced from Reference 45 (Figure 3, © 2003 Kluwer Academic Publishers), with kind permission of Springer Science and Business Media.

In addition to the extensive studies of native CDs described above, a number of studies of chemically modified CDs using coumarin guest fluorescence have also been reported [45,46,47,48,49]. Wagner *et al.* [45] used the fluorescence of included 7-methoxycoumarin (**7MC**) to compare the host properties of native β- and γ-CDs and their hydroxypropylated (HP-β- and HP- γ-CD) derivatives. A significant reduction in **7MC** fluorescence was observed in all four cases, as expected for this coumarin probe when experiencing a less polar environment. These results are illustrated in Figure 8, which shows fluorescence titration plots of the probe fluorescence intensity (F) as a function of the CD host concentration relative to that in the absence of CD (F_o_), and clearly shows the decrease in **7MC** fluorescence with increasing [CD].

Also shown in Figure 8 are the non-linear least-squares fit of the data to a 1:1 host-guest inclusion model to extract the binding constant K; relatively low values of K = 128 ± 32, 120 ± 20, 41 ± 8 and 40 ± 6 M^-1^ were obtained for β-CD, HP-β-CD, γ-CD and HP-γ-CD, respectively. Thus, the β-CD cavity provided a much better match for **7MC** than did the larger γ-CD cavity, as indicated by the larger binding constants. With β-CD, there was no observed difference in either the binding constant or the degree of fluorescence suppression when compared with the modified HP-β-CD; this lack of difference can clearly be seen by the overlap of these two fluorescence titration curves in Figure 8. This lack of difference between β-CD and HP-β-CD was in contrast to the results with other fluorescent probes studied by these authors, in which a significantly less polar cavity was experienced by probes included in HP-β-CD as compared to β-CD itself, due to the extension of the cavity by the alkylhydroxy groups. This lack of effect of the HP modifying groups indicated that **7MC** is well included within the β-CD cavity, and hence not affected by modifications of the CD rims. By contrast, there was a significant difference in the degree of fluorescence suppression (although not in binding constant) observed in HP-γ-CD as compared to γ-CD; in this case the much larger cavity allowed for interaction of the modifying groups with the included coumarin. Figure 8 provides a good illustration of the use of 7-alkoxycoumarin fluorescence titrations to extract the binding constant for inclusion. (In the case of 7-amino and other coumarin probes which exhibit fluorescence enhancement upon binding into a host cavity (such as the experiment illustrated as spectra in Figure 7), the resulting fluorescence titrations would look like mirror images of those in Figure 4 about the F/F_o_ = 1 line, *i.e.* a curved increase (fluorescence enhancement) which plateaus at higher host concentration.)

Bhattacharyya’s group extended their above-described studies on the solvation dynamics of **C153** in γ-CD to methylated β-CD, and again observed interesting and significant effects of the CD cavity on the solvation dynamics, with multiple kinetic components observed [46]. Velic *et al.* [47,48] used the fluorescence of coumarin probes to study the host properties of thiolated β-CD, which they subsequently attached to gold surfaces to construct fluorescent self-assembled monolayers. Most recently, Tablet and Hillebrand [49] used the inclusion of 3-carboxy-5,6-coumaric acid, a potential fluorescent marker for proteins, in native and HP-modified CDs as a model for its interactions with proteins. They observed a decrease in the coumarin fluorescence intensity, which they used to extract the binding constants for each CD. They also did molecular mechanics calculations to elucidate the structure of the CD:coumarin host:guest complex, and the contributions to the binding forces. 

A few other relevant studies of coumarins included within CD cavities will also be noted here to conclude this section. Chakraborty *et al.* [50] used both the steady-state and time resolved fluorescence of a number of 7-aminocoumarin guests to investigate the effect of CD inclusion on photoinduced electron transfer reactions, in this case to *N,N*-dimethylformamide. They observed a very strong retardation of the electron transfer rate by the CD at the high free-energy region for the electron transfer, which they explained using different binding possibilities for the coumarin in the CD cavity. In addition, a number of research groups have studied solid-state CD:coumarin complexes [51,52,53], although the fluorescence properties were not reported. 

It is clear from the wide range of studies described above that coumarin fluorescence has been successfully used to elucidate the physical and host properties of CDs, including the polarity and constriction of the internal cavity, the strength and nature of interactions between the CD and guest, and the effect of the CD host on guest reaction kinetics.

### Cucurbiturils

**Figure 9:**
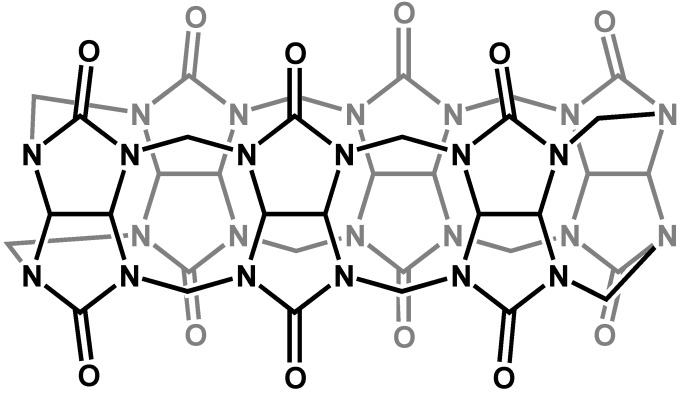
The cyclic structure of cucurbituril, CB[6].

Cucurbit[n]urils (CB[n]) are a family of macrocycles composed of n glycoluril units linked by two methylene bridges [54], as shown in Figure 9 for the parent (n=6) compound, cucurbituril. Compared to cyclodextrins, cucurbit[n]urils are extremely rigid, with well defined internal cavities, accessible through somewhat narrower carbonyl portals on both the top and bottom. The parent compound cucurbituril was first synthesized in 1904, but its structure and potential as a host compound were not elucidated until 1981 [55]. Since then, and particularly with the expansion of the family of hosts to include the n=5, 7 and 8 homologues in 2000 [56], cucurbit[n]urils have been the subject of growing interest and application.

There have been two reports on the inclusion of coumarin probes included within CB[n] hosts [57,58]. Nau and Mohanty [57] investigated the ability of CB[7] to both stabilize and enhance the fluorescence of a number of dyes, including coumarin **C102**. They observed a significant effect of CB[7] inclusion on the fluorescence properties of **C102**, including a blue-shift from 486 to 479 nm, and an increase in both the fluorescence lifetime and relative intensity, and explained this as being a consequence of the low polarizability inside the CB[7] cavity. Barooah *et al.* [58] studied the binding of a number of coumarin probes with the larger cucurbituril CB[8]. They found that most (but not all) of the coumarins formed dynamic inclusion complexes with CB[8], with varying stoichiometries, and could be made to undergo controlled photodimerizations within the CB[8] nanocontainers. They did not however report any fluorescence properties.

Compared to the extensive studies on the host properties of cyclodextrins, including the dynamics of the inclusion process itself as well as solvation within the CD cavity, the study of cucurbituril cavities using coumarin fluorescence remains vastly underexploited at this time.

### Other Molecular Hosts

There have been a scattering of fluorescence studies of coumarin host:guest inclusion complexes with molecular hosts other than CDs or CB[n]s. Frauchiger *et al.* [59] used coumarin **C102** as a dynamic probe of the local environment within amphiphilic starlike macromolecules (ASMs), which are essentially covalently-bound analogues of micelles (see next section). They have hydrophobic cores, which can encapsulate small hydrophobic molecules. The **C102** fluorescence results indicated that the ASM interior is quite polar, and has a high degree of heterogeneity. Both the solvent reorientation and guest diffusion rates were found to be significantly slower than in aqueous solution, and most importantly, approximately one-tenth of the **C102** guests were in microenvironments with significantly increased local friction. This latter result was used by the authors to indicate the potential use of these ASMs as drug carriers, with slow release of guests. This same research group also used coumarin fluorescence, in this case **C153**, to study related amphiphilic “scorpion-like” macrocycles (AScMs) as well as ASMs [60]. They used the fluorescence of encapsulated **C153** to determine the core polarity and local friction, and found significant differences between ASMs and AScMs. Finally, Shirota and Segawa [61] used the time-resolved fluorescence of **C153** to study the environment within crown ethers, as well as liquid oligoethylene oxides, and reported significant spectral shifting and reduced solvation times for the included **C153**.

Given the informative coumarin fluorescence-based studies of the cavity properties of CDs, CB[n]s and ASMs described above, there is significant (and as yet untapped) potential to use these coumarin fluorescent probes to study other discrete molecular hosts, such as calixarenes and cavitands. 

## Coumarins as Fluorescent Probes of Micelles

Micelles are spherical aggregates of surfactant molecules in solution. In aqueous solutions, the polar head groups form the micelle outer surface, with the organic tails oriented towards the interior, giving a relatively nonpolar core inside which hydrophobic molecules can become encapsulated. Micelles form when the surfactant concentration exceeds the *critical micelle concentration* (cmc). In organic solvents, the surfactants align in the opposite direction, giving *reverse micelles* with hydrophilic interiors. In a similar way as in the case of molecular hosts, fluorescent probes such as coumarins can be used to study the properties of micelles (such as the cmc, interior polarity and microviscosity) through measurement of micelle-induced changes to the probe fluorescent properties.

**Figure 10:**
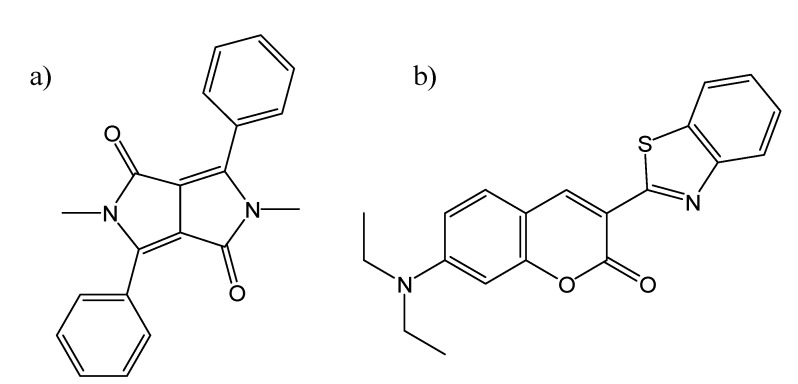
The chemical structures of the fluorescent probes a) DMDPP and b) C6.

There have been a number of survey studies using coumarin fluorescence to compare micelles based on different surfactants [62,63,64]. In two early studies published in 1994, Marques and Marques [62] used steady state and time-resolved fluorescence of a number of different coumarin probes with a wide range of micelles, to determine which coumarin species entered the hydrocarbon core of the various micelles, while Al-Kindy *et al.* [63] focused on a single ionic probe, coumarin-6-sulfonyl chloride (C-6SCl), in a series of anionic and cationic micelles. Dutt published an interesting paper addressing whether the microviscosity of micelles determined using fluorescence spectroscopy is probe-dependent [64]. He used two dissimilar probes, one non-dipolar (**DMDPP**) and the other the dipolar coumarin **C6**, encapsulated within the interior of six different types of micelles; these structures are shown in Figure 10. Significantly, he found almost identical microviscosities using these two probes for each micelle type, which justifies the experimental approach which is championed in this review article! 

In another comparative study, Hara *et al.* [65] looked at the pressure dependence of the solvation dynamics of coumarin **C153** (one of the most widely used coumarin probes for this purpose) in neutral TX100 (see below) as compared to anionic sodium dodecyl sulphate (SDS, see below) micelles. (They had previously reported in detail the pressure dependence of the solvation dynamics of **C153** in TX100 micelles [66,67].) They observed opposite pressure-dependent spectral shifts upon increased pressure in the two micelles, namely a blue-shift in TX100 and a red-shift in SDS. Concurrently, the solvation time was found to decrease in TX100 but increase in SDS; the authors attributed these results to the different hydration structures surrounding the micelles.

In addition to these studies by Hara *et al.* [65,66,67], a number of other groups have also reported coumarin fluorescence-based studies of Triton-X [68,69,70,71,72,73], making it by far the most extensively studied type of micelle using such techniques. Carnero Ruiz *et al.* [68,69,70] reported a series of papers on Triton X-100 (TX100) micelles. They used **C6** fluorescence depolarization to investigate the effect of the presence of KCl [68], ethylene glycol [69], and formamide [70] on the micelle formation, and found that electrolyte addition increased the microviscosity within the micelle whereas addition of formamide decreased it; these results were explained in terms of micellar solvation. In the case of ethylene glycol, however, solvation was not affected, but the micelle size or aggregation number decreased substantially. Kumbakhar *et al.* [71,72] used dynamic Stokes’ shift measurements of **C153** to study solvation dynamics in both TX-100 and TX-165 micelles. They found that TX-165 micelles have a much looser Palisade layer and lower microviscosity as compared to TX-100, which they attributed to differences in micellar hydration [71]. They also found that addition of LiCl significantly slowed the hydration dynamics in these micelles, due to strong hydration of the Li^+^ cations. Most recently, Sarkar’s group [73] studied microemulsions consisting of an ionic liquid and TX-100 micelles, using the fluorescence depolarization of **C153** and C151. They found that the solvent and rotational relaxation time of **C153** were not affected by addition of the ionic liquid, indicating that **C153** is located at the interface of the microemulsion, whereas the relaxation times of C151 were significantly increased upon increased fraction of ionic liquid, indicating that more C151 is located in the core of the microemulsions. Thus, coumarin fluorescence depolarization studies have been successfully employed to study the nature of TX-100 micelles. 

SDS micelles have also been widely studied using coumarin fluorescence [65,74,75,76,77,78]. Fery-Forgues *et al.* [74] were able to detect a sphere-to-rod structural transition in SDS micelles, using fluorescence changes in the same 4-hydroxycoumarin derivatives they used to investigate β-CD [41], described previously. Shirota *et al.* [75] used picosecond fluorescence spectroscopy of **C102** and **C153** to study the fast solvation and orientation dynamics within SDS micelles. De Paula *et al.* [76] used a benzoxazolyl coumarin to measure the polarity, microviscosity, and cmc of SDS micelles. Dutt [77] also used coumarin fluorescence anisotropy measurements (in this case **C6**) to study the microenvironments of SDS micelles, in the presence of various organic and inorganic salts. In a vastly different SDS system, Pantano *et al.* used the coumarin derivative C314 to study water/air interfaces containing SDS surfactants [78].

Other types of aqueous micelles which have been investigated using coumarin fluorescence include triblock copolymer micelles, which have been extensively studied by Kumbhakar *et al.* [79,80,81,82,83,84], Grant *et al.* [85,86] and Ghosh *et al.* [87] using **C153** as well as other coumarins; various alkyltrimethylammonium bromide micelles [88,89,90]; Tween 20 [91]; and Brij-35 micelles [92]. 

Reverse micelles, generated in organic solvents, have also been widely investigated using coumarin fluorescence [93,94,95,96,97,98,99,100,101,102,103,104,105,106,107,108]. Levinger *et al.* reported on the immobilization of water at the surfactant interfaces in reverse micelles using the coumarin derivative C343 [93], and subsequently published a review of ultrafast dynamics in such systems [94]. Raju and Costa [95,96] reported a series of studies of Aerosol OT (AOT) reverse micelles, using the coumarin derivatives **C35** (Figure 2b) [95], as well as C480 and a water-insoluble aminocoumarin derivative [96]. Sarkar’s group [97,98,99,100,101] have published extensively on methanol and acetonitrile reverse micelles, using C490 [97]; **C152A** (Figure 2a) [98]; **C153** [99]; and other coumarin dyes [100,101]. They consistently found that the probe solvation time is strongly dependent on the ratio of polar solvent to surfactant concentration in the case of methanol, but not in the case of acetonitrile, and explained this observation in terms of the presence or absence of hydrogen bonding. A number of other papers have also been published on AOT reverse micelles studied using coumarin fluorescence properties [102,103,104,105,106], as well as on AOT micelle films [107,108].

## Coumarins as Fluorescent Probes of Polymer Hosts

Guest molecules can become included within the folds or pockets of polymers in solution, or within polymer thin films. Trenor *et al.* [109] published an excellent comprehensive review in 2004 of the use of coumarins both to study the properties of polymers using fluorescence studies, and to prepare polymers with useful optical properties, such as light harvesting. This current review will therefore only cover articles on the use of coumarins as fluorescent probes of polymer hosts which have been reported since that 2004 review. 

Prabhugouda *et al.* [110] used the coumarin derivative C515 as an acceptor molecule to study energy transfer between dopants within polystyrene (one of the most important polymers worldwide) in aqueous solutions. Corrales *et al.* [111] also studied energy transfer processes within a coumarin-doped polymer, namely poly(ethylene terephthalate). In this case, the coumarin derivative C337 was used as the donor, and energy transfer to the polymer itself resulted in a strong enhancement of the polymer chemiluminescence. This emission was used to study the properties of the polymer, as it was found to be sensitive to the polymer morphology (including crystallinity) and probe mobility within the polymer. 

A number of researchers have used coumarins to investigate the properties of polymer thin films [112,113,114]. Mason *et al.* [112] doped a polymer photoresist film with the pH-sensitive coumarin probe **C6**. They were able to measure the relative fluorescence signals from the neutral and protonated forms of C6 to determine the range of acidity and inhomogeneity within the polymer films, and to determine that proton exchange within these films happens very slowly below the glass transition temperature. Frenette *et al.* [113] also used **C6** to study polymer resist films. They were able to determine the catalytic chain length of the prepared PMMA thin films using the coumarin fluorescence. Finally, Oh *et al.* [114] prepared a polymerizable coumarin derivative, and prepared fluorescent polymers with a range of practical applications in latex films. 

## Coumarins as Fluorescent Probes of Solid Host Materials

Two different types of solid hosts incorporating coumarin guests can be distinguished. Porous solids, such as zeolites, contain permanent cavities or channels into which guest molecules can become included. Glassy or crystalline solids, however, require that the guest be included in the solid formation process, such as crystallization, annealing or sol-gel techniques. The major difference between these two types of solid hosts involves the dynamics of the inclusion process. In the case of porous solids, a dynamic equilibrium between the included and free guest is established, much like the case of hosts in solution as described above. The binding constants are usually much higher than in solution, so that the guests are more effectively trapped, but they can still be removed. In nonporous crystalline or glassy solids, however, the guests are effectively a permanent part of the structure. This review will focus on both of these two major types of solid hosts, with sol-gel glasses being the primary type of nonporous host which has been studied using coumarin fluorescence.

### Porous Solid Hosts

A number of studies have been reported using coumarin fluorescence to characterize the internal pores and channels of zeolites and related porous aluminosilicate structures. Corrent *et al.* [115] studied the acid-base properties of **C6** (widely used as a pH-sensitive probe as described previously) within various zeolites. They were able to show that the commonly used faujasite zeolite NaY, which is generally considered to be nonacidic, in fact has acidic sites within its heterogeneous structure. In two related faujasite zeolites, HY 100 and CBV 740, the dication of **C6** was observed, which was explained by the very high Bronsted and Lewis acidity, respectively, of these two zeolites. Kamijo *et al.* [116] used **C153** fluorescence to characterize synthetic silica nanochannels prepared inside the pores of an anodic alumina membrane. They used time-resolved fluorescence to measure the solvation relaxation times of **C153** co-included with various alcohols in the nanochannels; these relaxation times were found to be much longer than in bulk alcohol solvent, but that changing the bulkiness of the alcohol (*e.g.* decanol *vs.* ethanol) had no significant effect. They concluded that the alcohols are rigidly held in the silica nanochannels through an extended hydrogen bonding network.

Other porous solids have also been investigated using coumarin fluorescence, including MCM-41 and Ti-MCM-41 mesoporous molecular sieves [117,118], which were found to have a pore size-dependent effect on the fluorescence of a number of coumarin derivatives; a pillared layer clay nanocomposite [119], which was found to enhance the fluorescence of **C1**; and anodic aluminum oxide films with coumarin 7 embedded in the pores [120], which exhibited an additional, long wavelength coumarin emission band.

### Coumarin-Doped Glasses and Crystals

Sol-gels and related materials have been by far the most widely studied example of coumarin doped glasses. The sol-gel process is a synthetic procedure which allows for the preparation of glasses and other materials at room temperature. The preparation and properties of fluorescent probe- (including coumarin) doped sol-gel glasses [121] and nanocomposite materials [122] have been previously reviewed; therefore, only brief overview of the use of coumarin fluorescence to study the properties of such glasses will be presented here.

Takahashi *et al.* used coumarins C4 and **C6** to study amorphous silica sol-gel glasses [123] and a sol-gel coating film [124], respectively. In both cases, significant red-shifted coumarin emission was observed, related to the probe acid-base properties. Oh *et al.* also used coumarin C4, in this case to probe the properties of a silica-PDMS xerogel [125]. Ferrer *et al.* [126] used **C153** to measure the microviscosities in silica gel-glasses, while Baumann *et al.* [127] used this same coumarin dye to study the effects of confinement in ethanol within a sol-gel glass on the probe’s rotational and solvation dynamics. Other coumarins which have been studied within sol-gel glasses include C2 [128]; C152 [129]; C307 [130,131]; and silylated coumarin dyes [132,133,134]. 

Reports on the fluorescence of coumarins doped in crystalline solids have been much fewer. Ganschow *et al*. [135] used coumarin C40 doped in AlPO_4_-5 single crystal molecular sieves to investigate their properties, and found that the coumarin dye was uniformly distributed throughout the single crystals, and that these crystals had good optical properties for potential photonics applications. Similarly, Galian *et al.* [136] doped coumarin **C6** into Photonic Crystal Fibers (PCFs) to investigate their properties and potential photonics applications.

### Other Solid Host Materials

Coumarin fluorescence has also been used to study a range of other types of solid hosts, which do not fit under either of the above two categories. Aloisi *et al.* [137] intercalated coumarin-3-carboxylic acid with other donors and acceptors between the layers of Mg-Al hydrotalcite-like compounds to generate nanocomposite materials, and used the emission to characterize the materials themselves as well as energy transfer processes between the intercalated probes. Similarly, Fujii *et al.* [138] co-intercalated coumarin probes with rhodamine 6G within the layers of a novel luminescent layered material, and used the coumarin fluorescence intensity to monitor the energy transfer to the rhodamine 6G probes. Other interesting solid materials probed using coumarin fluorescence include cellulose derivatives [139] and titania-based self-cleaning materials [140].

## Other Heterogeneous Systems

In addition to the molecular, micellar, polymer and solid host systems described in detail above, there have been a few coumarin-fluorescence-based studies of other types of heterogeneous host systems. These systems include Langmuir-Blodgett films [141,142]; self-assembled monolayers [143] and polyelectrolyte multilayer nanocontainers [144].

## Summary

Coumarins as a family of compounds represent one of the most versatile and applicable family of fluorescent probes, with a wide range of sizes and hydrophobicity. Even more importantly, coumarin fluorescence shows a very broad range of responses and sensitivity to various properties of the local environment, including polarity, polarizability, microviscosity, hydrogen bonding potential and pH. 

Coumarins exhibit fascinating and unique photophysical and spectroscopic properties; different derivatives can show different or even opposite behavior upon the same change in conditions. Most strikingly, 7-aminocoumarins and 7-alkoxycoumarins exhibit opposite polarity dependence: 7-aminocoumarins show highest fluorescence intensity in nonpolar media, whereas 7-alkoxy show highest fluorescence intensity in polar media. This opposite behaviour is a result of very different underlying photophysics, and in particular different major nonradiative decay pathways for these two types of coumarins, namely TICT *vs.* ISC. Furthermore, this opposing polarity dependence means that a specific coumarin probe can be chosen to show a desired fluorescence change, for example either “switch on” (increased fluorescence) or “switch off” (decreased fluorescence) behavior upon inclusion in a specific cavity or region of a heterogeneous system.

A number of specific coumarin derivatives have been particularly exploited due to their unique physical and spectroscopic properties. For example, **C153**, a rigid 7-aminocoumarin, has been widely applied as a probe of solvation dynamics, as the lack of intramolecular rotation makes it particularly sensitive to solvent reorientation. Another example is **C6**, which is a pH-sensitive probe with vastly different fluorescence emission properties in its neutral and protonated form; this probe has been extensively used to investigate the acid-base properties within heterogeneous systems. 

It is clear from the extensive list of studies described in this review that coumarin fluorescence can and has been used quite effectively as a probe of the physical, structural and chemical properties of a wide range of heterogeneous host systems, including molecular hosts, micelles, polymers, and porous, glassy, and crystalline solids. Researchers studying such systems should be aware of the tremendous applicability of coumarin probe fluorescence, and the extensive information which can be obtained from their use. Furthermore, as noted in various sections, coumarin fluorescence could potentially be used for other, as yet explored molecular hosts and heterogeneous systems, and should be considered as an option for all researchers preparing and investigating novel inclusion hosts and materials.
